# Peripersonal Space and Bodily Self-Consciousness: Implications for Psychological Trauma-Related Disorders

**DOI:** 10.3389/fnins.2020.586605

**Published:** 2020-12-10

**Authors:** Daniela Rabellino, Paul A. Frewen, Margaret C. McKinnon, Ruth A. Lanius

**Affiliations:** ^1^Department of Psychiatry, Western University, London, ON, Canada; ^2^Imaging Division, Lawson Health Research Institute, London, ON, Canada; ^3^Department of Psychology, Western University, London, ON, Canada; ^4^Mood Disorders Program, St. Joseph’s Healthcare, Hamilton, ON, Canada; ^5^Department of Psychiatry and Behavioural Neurosciences, McMaster University, Hamilton, ON, Canada; ^6^Homewood Research Institute, Guelph, ON, Canada

**Keywords:** peripersonal space, bodily self-consciousness, trauma-related disorders, neurobiology, defense response, multisensory processing, PTSD, dissociation

## Abstract

Peripersonal space (PPS) is defined as the space surrounding the body where we can reach or be reached by external entities, including objects or other individuals. PPS is an essential component of bodily self-consciousness that allows us to perform actions in the world (e.g., grasping and manipulating objects) and protect our body while interacting with the surrounding environment. Multisensory processing plays a critical role in PPS representation, facilitating not only to situate ourselves in space but also assisting in the localization of external entities at a close distance from our bodies. Such abilities appear especially crucial when an external entity (a sound, an object, or a person) is approaching us, thereby allowing the assessment of the salience of a potential incoming threat. Accordingly, PPS represents a key aspect of social cognitive processes operational when we interact with other people (for example, in a dynamic dyad). The underpinnings of PPS have been investigated largely in human models and in animals and include the operation of dedicated multimodal neurons (neurons that respond specifically to co-occurring stimuli from different perceptive modalities, e.g., auditory and tactile stimuli) within brain regions involved in sensorimotor processing (ventral intraparietal sulcus, ventral premotor cortex), interoception (insula), and visual recognition (lateral occipital cortex). Although the defensive role of the PPS has been observed in psychopathology (e.g., in phobias) the relation between PPS and altered states of bodily consciousness remains largely unexplored. Specifically, PPS representation in trauma-related disorders, where altered states of consciousness can involve dissociation from the body and its surroundings, have not been investigated. Accordingly, we review here: (1) the behavioral and neurobiological literature surrounding trauma-related disorders and its relevance to PPS; and (2) outline future research directions aimed at examining altered states of bodily self-consciousness in trauma related-disorders.

## Introduction

Peripersonal space (PPS) refers to the space surrounding the body where we can reach or be reached by external entities, including objects or other individuals (Rizzolatti et al., 1997; Brozzoli et al., 2012b). It is a fundamental characteristic of our everyday life, where we move through space to reach our goals, interact with other individuals, avoid colliding with objects or other people, and act in a way that protects our bodies from potential incoming threats (de Haan et al., 2016). In order to engage in such a complex task, we need to integrate visual, auditory, tactile, interoceptive, and proprioceptive stimuli from our own body and from the environment, thereby not only constantly monitoring our body location in space but also scrutinizing changes occurring in the surrounding space (Salomon et al., 2017). PPS thus represents an aspect of bodily self-consciousness that emerges and changes with the flow of experience (Noel et al., 2015b, 2018b). Interestingly, PPS can also be viewed as a “defensive zone,” activating bodily alarm reactions when PPS boundaries are surpassed or violated (Holmes and Spence, 2004; Graziano and Cooke, 2006; Sambo et al., 2012; de Haan et al., 2016). Accordingly, the investigation of PPS is critical to the study of psychopathology, in particular anxiety disorders such as phobias and trauma-related disorders, where fear responses and/or altered states of bodily self-consciousness play a crucial role. A substantial number of studies provides further evidence of the crucial role played by PPS in everyday experience. Taken together, these studies have identified a neural network in both animal models and humans that is dedicated specifically to bodily self-consciousness and PPS representation (Rizzolatti et al., 1997; Fogassi et al., 1999; Gallivan et al., 2011; Brozzoli et al., 2012a; Blanke et al., 2015; Cléry et al., 2015; di Pellegrino and Làdavas, 2015; Limanowski and Blankenburg, 2015).

In this review, we discuss PPS and its relation to bodily self-consciousness in psychopathology using a multidisciplinary perspective that includes cognitive science, neuroscience, psychiatry, and clinical psychology. We present first a definition of PPS and illustrate its characteristics and functions. We then identify the cortical and subcortical structures underlying PPS representation in animal models and in humans. Furthermore, we explore the scant literature describing the relation between psychopathology and PPS, with a focus on trauma-related disorders, where out-of-body experiences can be an important component of the underlying psychopathology. Finally, we describe the importance of studying PPS in trauma-related disorders in an effort to not only enhance our understanding of the critical role of bodily self-consciousness in these disorders but also to deepen our insight regarding the characteristics of PPS that are influenced by psychological traits and states.

## Peripersonal Space and Bodily Self-Consciousness

Bodily self-consciousness has been described as the conscious experience of owning a body (self-identification), occupying a specific location in space (self-location), and having a specific perspective from where to perceive the world that coincides with one’s body (first-person perspective; Blanke, 2012). While all these aspect of bodily self-consciousness are interconnected to create a sense of self that is embodied, they are also related to the representation of the space surrounding the body (PPS). Here, body ownership has been largely studied using experimental paradigms that aim to create the illusion of owning external body-parts or alter bodies that are located within the PPS, suggesting the PPS as a spatial constraint for the body ownership illusion to occur (Botvinick and Cohen, 1998; Makin et al., 2008; Ehrsson, 2012; Serino, 2019). In addition, self-location is an essential reference to build the PPS representation (since the PPS surrounds the body) and has been found to depend strongly on the first-person perspective (Blanke, 2012). Neuroimaging studies have corroborated the interaction of these different aspects of bodily self-consciousness, where overlap has been identified among the neural correlates of body ownership, self-location, first-person perspective, and PPS (see paragraph “Neural Correlates of Peripersonal Space in Humans” on neuroimaging studies in humans).

Multisensory integration of information represents a crucial mechanism underlying all aspects of bodily self-consciousness. Park and Blanke (2019) have recently proposed interoceptive-exteroceptive bodily self-consciousness (xBSC) as a conceptualization that aims to include both exteroceptive bodily self-consciousness (gathering information within the PPS) and interoceptive bodily self-consciousness (gathering information from the inner body) into a unique integrated neural system (for details please see Park and Blanke, 2019). In this manuscript, we focus our attention on PPS specifically, while considering the dynamic interplay of PPS with other aspects of the conscious experience that build a fluid embodied sense of self (body ownership, interoception, self-location, and first-person perspective).

### Peripersonal and Extrapersonal Space

Peripersonal space (PPS) is defined as the space surrounding the body where we can not only reach and manipulate objects by movement but we can also be reached by external elements, including other individuals. By contrast, extra-personal space constitutes an area further from the body, where objects cannot be reached, and the environment is explored primarily through visual means (Bartolo et al., 2014; di Pellegrino and Làdavas, 2015). Interestingly, this dichotomous (in-or-out) conceptualization of PPS has been debated recently by Bufacchi and Iannetti (2018). They suggest, instead, that the PPS unfolds as a set of graded fields, similar to magnetic fields, where the representation of the PPS depends on its relevance within a specific context and the measures used to define it. Taken together, these notions suggest that PPS representation depends heavily on the relevance of possible actions aimed at creating or avoiding contact between the body and external elements. Further studies are needed to unravel this complexity.

The concept of PPS stems from electrophysiological research on monkey models (Hyvärinen and Poranen, 1974; Mountcastle, 1976; Leinonen and Nyman, 1979; Rizzolatti et al., 1981b). Here, researchers identified a specific population of neurons in the fronto-parietal cortex−dedicated to sensorimotor processing−that were specifically activated by stimuli presented in the space surrounding the animal; these neurons were bimodal (visuo-tactile) and responded selectively to somatosensory stimuli. These neurons may underlie bodily self-consciousness in that they are dedicated specifically to the integration of somatosensory bodily signals and stimuli originating in a limited space surrounding the body.

### Peripersonal Space and Body Schema

Movement of the body in space while monitoring its surroundings is critical to the notion of PPS (Holmes and Spence, 2004). As PPS facilitates guidance of the body through space, it requires integration of an individual’s internal bodily representation and body schema with the surrounding space (Holmes and Spence, 2004; Cardinali et al., 2009). Body schema has been defined as the active and operative non-conscious performance of the body in relation to the environment and is thought to integrate multisensory information derived from proprioceptive, interoceptive, somatosensory, visual, and auditory input from the body and the environment (Gallagher, 1986). For example, it reflects and determines the posture of the body in the environment. Critically, the integration of body schema and PPS allows bodily self-consciousness to emerge with reference to a physical body that is perceived as one’s own body in a specific location in space (self-identification and self-location) (Blanke, 2012; Noel et al., 2015b, 2018b). The relationship among plasticity, multisensory integration in PPS, and body schema representations has given rise to the question whether the two representations correspond in fact to the same concept (Cardinali et al., 2009). While a definitive conclusion has not been achieved yet, Cardinali et al. (2009) have argued that the two representations should be considered dissociable in principle (one can change without modifying the other), both in terms of spatial continuity (body schema is constrained within the body limits) and time (different latency in plastic changes). Further studies have also supported the notion of two separate mechanisms underlying the two constructs (Bassolino et al., 2014; D’Angelo et al., 2018).

### Peripersonal Space Extension

To identify and investigate PPS, researchers have focused on the mechanisms underlying multisensory integration. Multimodal neurons involved in the representation of PPS activate when tactile, visual, and auditory stimuli are presented close to the body but not when far away from the body, thus allowing for the identification of PPS boundaries around the body. Critically, researchers have recognized approaching stimuli as most relevant to identify PPS boundaries, likely because they might represent a potential incoming threat (Graziano and Cooke, 2006). Here, a research study found that an approaching auditory stimulus increased the processing of a concomitant tactile stimulus on the body surface when the auditory stimulus was perceived at a limited distance from the hand, thus setting the boundaries of PPS (measured through reaction time to a tactile stimulus to the hand delivered at different temporal delays from the sound onset, where the sound was delivered in a way that created a looming effect via volume modulation; Canzoneri et al., 2012). It is also important to note that this effect was stronger for an approaching auditory stimulus as compared to a receding one. Similarly, Noel et al. (2015b) identified PPS boundaries through reaction times in response to vibro-tactile stimuli administered to the participant’s chest while task-irrelevant sounds, administered through loud speakers disposed at gradually closer distances, loomed toward the subject’s body. Reaction times increased when the boundaries of PPS were surpassed by the auditory stimuli. Taken together, these results demonstrate that during multisensory processing we assign unique relevance to external stimuli that are closer to the body and dynamically looming toward the body.

### Peripersonal Space Is Body-Part Centered

Research in humans and animal models have shown that PPS can vary depending on what part of the body is examined. Indeed, a specific representation of PPS has been identified for the hand, the arm, the face, and the trunk (Serino, 2019). This unique characteristic accounts for the dynamic reshaping of PPS representations when body parts move in space while the rest of the body remains still. For example, when researchers investigated PPS in relation to the hand, they found that when the subject moved her/his hand, the PPS would update to follow the movement, thus maintaining PPS map coordinates centered on the hand (Brozzoli et al., 2012a; di Pellegrino and Làdavas, 2015; Dijkerman and Farnè, 2015; Serino et al., 2015b). This characteristic is maintained through integration between the visual/auditory receptive fields and the somatosensory receptive fields within bimodal neurons in the fronto-parietal regions (Graziano et al., 1997b). In a seminal series of seven studies, Serino et al. (2015b) investigated PPS in healthy humans, concluding that PPS appears to involve at least three differentiated representations centered in the hands, the face, and the trunk. Moreover, these representations appear to be unified by a common reference frame of the trunk. For example, when an individual moves one of her/his hands, the relative PPS also modifies according to the new location of the hand in space. However, the extent of PPS boundaries relative to the hand depends on the proximity to the trunk, appearing larger when the hand is closer to the trunk. Serino and colleagues suggest this effect to stem from the trunk-centered PPS taking over the hand-centered PPS when the hand is very close to the trunk. Moreover, the extent of PPS among individuals tested appeared increasingly larger for the hands (average 45 ± 7 cm), the face (average 59 ± 6 cm) and the trunk (average 72 ± 7 cm), respectively. Accordingly, peri-trunk personal space is assumed to best represent a whole-body reference frame for the egocentric representation of self (Serino et al., 2015b). Finally, these studies revealed that whereas receding stimuli modulate the PPS for the hands, no such effect was observed for the face and trunk.

In summary, the PPS representation is characterized by: (a) being specifically dedicated to a limited space surrounding the body; (b) being hyper-sensitive to stimuli that are moving toward the body; (c) being anchored to body parts (hand-centered, face-centered, arm-centered, and trunk-centered PPS have been identified so far). Collectively, these findings assist in shaping our understanding of PPS as an essential feature for bodily self-consciousness, where either distinct and/or unified flexible representations of our own body in space guide our actions through the external environment.

## Plasticity of Peripersonal Space

A key feature of PPS is its plastic and dynamic representation. Whereas plastic changes refer to the flexibility elicited through training or learning, dynamic changes occur in response to modifications in the environment or in response to the internal state changes of an individual, including emotional state changes (Cléry et al., 2015). Extensive animal and human research has demonstrated flexibility of the PPS (Holmes and Spence, 2004; di Pellegrino and Làdavas, 2015; Dijkerman and Farnè, 2015). For example, plastic changes have been demonstrated in studies where repeated use of tools (e.g., a stick) resulted in remapping of PPS representation, where the tool was perceived as an extension of the body (e.g., the hand) and was incorporated within the hand-centered PPS (Berti and Frassinetti, 2000; Bonifazi et al., 2007; Làdavas and Serino, 2008). Berti and Frassinetti (2000) seminal study showed remapping of PPS in an individual with left visual neglect following right hemisphere stroke. Here, the patient’s neglect involved a dissociation between near and far space, where neglect was apparent in the “near space” only. The use of a tool (a stick in one hand) was able to change the neglect manifestation, as the “far space” was remapped as “near space” in the patient’s brain, and neglect now appeared in what was previously coded as “far space.” In another recent study, plastic changes were associated with the body modifications occurring in the third trimester of pregnancy: the PPS of the expectant mothers became enlarged over time, incorporating more gradual boundaries between near and far space as measured by an audio-tactile integration task (tactile stimulation to the abdomen concurrent with looming sounds delivered via loudspeakers, which were positioned in the far space − 1 m away from the participant’s hand − and the near space − close to the hand − in order to give the perception of a sound traveling toward the participant’s body; Cardini et al., 2019).

This gradual expansion of the boundaries between near and far space is of critical importance in psychological disorders involving altered perception of self-other distinction and has indeed been observed in schizophrenia and autism spectrum disorders (Noel et al., 2017). Here, Noel and colleagues (Noel et al., 2017) proposed that whereas shallow PPS boundaries (as revealed by a larger space indexing the transition from extrapersonal to peripersonal space) reflect a weaker self-other distinction, steep PPS boundaries (smaller space indexing the transition from extrapersonal to peripersonal space) point toward a rigid self-other differentiation (Noel et al., 2017). Taken together, these studies suggest a gradual passage from PPS to extrapersonal space (and vice versa), where PPS boundaries are conceptualized in terms of gradients instead of a dichotomous in-out transition. Interestingly, these studies demonstrate further how psychological characteristics may affect PPS size, as well as the graduality of PPS boundaries.

Body illusion experiments have also shown dynamic changes in PPS as revealed through illusions experienced by subjects. For example, when a virtual body was placed *in front* of subjects and was stroked in synchrony with the real body, Noel et al. (2015b) reported a self-location drift toward the virtual body. Accordingly, the boundaries of PPS extended in the front space toward the virtual body and shrunk in the back. This outcome was interpreted as a shift in PPS representation from being centered on the physical body to being centered on a new subjectively experienced location of the self (Noel et al., 2015b).

The use of a mirror has also been shown to produce dynamic changes in PPS in humans. For example, Maravita et al. (2000, 2003) investigated cross-modal extinction in a patient with a right-hemisphere lesion, where a visual stimulus delivered to the right side of the visual field impaired the perception of a simultaneous tactile stimulus on the left side of the body. This extinction of touch to the left hand occurred in particular when a simultaneous visual stimulus (flashlight) was delivered close to the right hand (thus within PPS). Interestingly, the same outcome resulted (touch extinction to the left hand) when the patient saw the visual stimulus close to his right hand as a reflection in a mirror placed directly in front of him/her. Importantly, the same result was not obtained when the mirror was removed and both the visual stimulus and the hand (a fake hand reproducing the patient’s hand) were distant in space (thus in extrapersonal space). These results demonstrated that the space surrounding one’s body reflection in a mirror is perceived as PPS.

Similar results were obtained among healthy participants in a study investigating event-related potential (ERP) correlates of crossmodal interactions between tactile (delivered via a tactile stimulator) and mirror-reflected visual stimuli (LED light) (Sambo and Forster, 2011). Here, multimodal stimuli (in this case, tactile, and visual) produced enhanced ERPs within the somatosensory cortex when the stimuli were spatially congruent (on the same side with respect to the body) and close to the body (the visual stimulus was delivered within the PPS) as compared to when the stimuli were spatially incongruent. These results using mirror-reflected visual stimuli replicate findings observed when a visual stimulus was delivered near the actual hand of the study participant (thus within the PPS). Taken together, these studies indicate that when our body is reflected in a mirror, images appearing close to our reflected body are perceived as pertaining our PPS, with the PPS representation dynamically remapping to include a space that would otherwise be considered to be in the extrapersonal space. Even body shadows have been found to initiate a similar remapping of PPS. When the body shadow becomes part of the body schema, the representation of PPS is reorganized accordingly (Pavani and Castiello, 2004).

Another impressive finding by Noel et al. (2015a) revealed remapping of the PPS during walking as compared to a standing position. Here, the researchers tested PPS boundaries relative to the trunk through audio-tactile stimulation (tactile stimulation of the chest during approaching sound stimuli) and showed that PPS extension, measured via response times to the tactile stimulation, clearly increased in the walking (around 166cm PPS extension) as compared to the standing condition (65 to 100 cm PPS extension). This outcome confirms further the dynamic nature of PPS, reshaping continuously in relation to body movements and to the relevance of its surroundings. This integration of body location and objects in the environment, essential to bodily self-consciousness, appears to require an extended PPS when moving through space. Moreover, the relevance of the body’s surroundings appears to increase during walking, finding that could stem, in part, from increased attention to potential new encounters or new obstacles in the space while we move.

Interestingly, recent studies have utilized biologically plausible network modeling to simulate and investigate the neural networks underlying the PPS representation (Serino et al., 2015a; Noel et al., 2020). Here, Hebbian learning strengthening and weakening of feedback and feedforward synapses have been used to unravel plasticity of PPS. For example, the neural network model used in the work presented by Serino et al. (2015a) was able to predict the extension of the PPS representation without tool-use and was subsequently confirmed by behavioral measurements. A similar computational approach led the authors to suggest that neural adaptation could account for dynamic aspects of PPS, such as PPS resizing at the speed of looming stimuli (Noel et al., 2018a).

Notably, equally dynamic social cognitive processes and interactions with other people may affect the extent of PPS. In a study investigating PPS and social interactions, face-centered PPS appeared to shrink in the presence of another person as compared to a mannequin placed at a distance of 1m from the participant (Teneggi et al., 2013). Specifically, when the study participant was in the presence of another person, PPS boundaries moved closer to the participant (thus the PPS size shrank) as compared to when the study participant was in the presence of a mannequin and no such effect occurred. Interestingly, however, a larger PPS was observed in the presence of another person considered cooperative (previously showed to be cooperative with the subject during an economic game) as compared to the presence of a non-cooperative other individual (Teneggi et al., 2013). Taken together, these findings suggest that PPS functions serve not only as an interface between internal sensorimotor information and the spatial environment needed to grasp or manipulate objects, but also play a critical role in higher-order social cognitive processes and in interpersonal relationships.

## Functions of Peripersonal Space

### Motor Function

As Holmes and Spence (Holmes and Spence, 2004) pointed out, PPS guides us through space, integrating different perceptive modalities (visual, tactile, auditory, interoception, and proprioception) to allow us to locate ourselves in space and to move through it safely. Indeed, PPS has been defined by the motor function of reaching, grasping, and manipulating objects in our surroundings (Brozzoli et al., 2012b). This view is supported further by observations of the plasticity characterizing the PPS, as exemplified by remapping of the PPS during body movement. Here, the PPS updates its representation while motor action occurs (Brozzoli et al., 2012b), an observation exemplified in studies investigating PPS during walking or during limb movement (di Pellegrino and Làdavas, 2015; Noel et al., 2015a). For example, Brozzoli and colleagues (Brozzoli et al., 2011) reported that visuo-tactile interference occurs during voluntary action performed by grasping a target object, thus providing evidence for a remapping of PPS during movement of the hand^1^.

More recently, a series of studies (Dijkerman and Farnè, 2015) examined the bidirectional association between PPS and motor action, where PPS is modulated by motor action and influence activity within the motor system. Here, one study revealed that keeping one arm at forced rest for a prolonged time (10 h) resulted in a decreased PPS around that same arm (Bassolino et al., 2014). Furthermore, using transcranial magnetic stimulation while recording motor evoked potentials, Finisguerra et al. (2015) found that auditory stimuli within the PPS (within 60 cm from the subjects’ hand) can modulate neural activity within the motor system (motor excitability; Finisguerra et al., 2015), thus providing further evidence for the important relation between motor action and PPS.

### Defense Zone

Central to work surrounding PPS is the notion that this space represents not only a zone where an individual can have an effect on objects, but also the space where we can be reached by external entities (objects, animals or people) and potentially be harmed. As PPS surrounds one’s body, it represents a vital space for any contact with the external environment. This perspective has been adopted by researchers investigating PPS as a defense zone. For example, de Haan et al. (2016) found that PPS was modulated by visuo-tactile predictions of an incoming threat: when subjects saw the image of a spider approaching their hand, their response time to a contingent tactile stimulus to the hand (time employed to press a foot pedal after perceiving a tap on the hand) was significantly faster. Critically, this outcome was apparent only for participants afraid of spiders. In a similar experiment, participants with a fear of dogs showed larger PPS boundaries (audio-tactile integration task) when in the presence of a rear-approaching sound that resembled a growling dog as compared to non-fearful participants (Taffou and Viaud-Delmon, 2014). Interestingly, studies on monkeys have shown that when neurons in the fronto-parietal cortex involved in the PPS representation (polysensory zone) are stimulated, a defensive arm movement is triggered, thus supporting the defensive purpose of the PPS at the neural level (Cooke and Graziano, 2003).

The hand-blink reflex has also served as a key experimental procedure to study the concept of PPS as a defense zone. This reflex consists of involuntary blinking of the eye that is generated by stimulation of the median nerve in the wrist and is mediated by the reticular formation in the brainstem (Miwa et al., 1998). In a groundbreaking experiment using the hand-generated blink reflex, Sambo et al. (2012) demonstrated that the amplitude of the blink reflex is mediated by the distance between the hand itself and the face, thus supporting the notion of the PPS as a defensive zone. Specifically, the blink reflex occurred only when the stimulated hand was located within the face-centered PPS, that is, the PPS representing the space surrounding the face. In a further study, Fossataro et al. (2016) investigated the influence of social interactions on the hand-blink reflex, showing that interpersonal interaction can affect subcortical defensive responses as measured by the hand-blink reflex (increased blink reflex during stimulation of the median nerve in the wrist in the study participant when someone else’s hand enters the individual’s PPS). Notably, this effect was dependent on the level of empathy endorsed by study participants, with an increased defensive blink reflex observed in persons with higher empathy scores as measured through the Interpersonal Reactivity Index − IRI; (Davis, 1983). Critically, these findings suggest that brainstem circuits involved in the hand-blink reflex are modulated in a top-down fashion by neocortical brain areas involved in not only proprioceptive location of body parts in space but also in social cognition.

Additional work suggests that the defensive function of PPS depends on attention selection (de Haan et al., 2016), where a visually detected threat appears to bias our attention toward the imminent potential harm, particularly when the threat is approaching the body and thus has the potential to enhance response to any following stimulus in that particular zone (Poliakoff et al., 2007; Carretié et al., 2009; Ferri et al., 2015). This putative defensive mechanism appears conserved and further amplified in psychological disorders that involve attention bias and threat responding, such as trauma-related and anxiety disorders. For example, a study investigating freeze-like response (characterized by tonic immobility of the body and tested as a slowing of reaction times) to approaching stimuli (thus impinging on PPS) in humans showed that reaction times were significantly slower during exposure to a threatening as compared to a non-threatening stimulus and that this response correlated with state-anxiety scores endorsed by participants (Sagliano et al., 2014).

### Dual Model

de Vignemont and Iannetti (2015) have proposed a dual model of PPS, where a goal-oriented PPS can be differentiated from a defensive PPS by their purpose and sensorimotor features. Here, goal-oriented PPS serves the purpose of intentionally moving body parts (primarily arms and hands) to reach a goal such as grasping and manipulating objects. At the sensory level, the motor-oriented PPS would be focused primarily on fine-grained features of objects in order to better execute reaching and grasping actions. By contrast, a defensive PPS would be characterized by automatic protective actions, such as avoiding contact with objects, or by no actions at all (e.g., freezing/tonic immobility states). Moreover, it is postulated that this defensive PPS encompasses the entire body and would be influenced by the salience of an incoming stimulus, where a potential threat would represent the most salient information in the surrounding environment that needs to be rapidly detected for survival. This defensive PPS is also expected to be characterized by sharp boundaries. Here, studies using the hand-blink reflex as a measure of the defensive response found a sharp change in the defensive response when a hand entered the PPS, thus providing support for sharp boundaries characterizing the defensive PPS (Sambo and Iannetti, 2013). Although compelling, the dual model hypothesis has yet to be supported by empirical studies.

In summary, two main functions of the PPS have been identified: (1) a goal-oriented representation of space where bodily self-consciousness subserves motor intentions and action planning; and (2) a defense zone where bodily self-consciousness is oriented to protect the body from incoming potential threats.

## Neural Correlates of Peripersonal Space

### Neural Correlates of Peripersonal Space in Animal Models

The initial definition of PPS derived from neurophysiological studies performed in primates in the 1980s examining bimodal neurons in the periarcuate (premotor) cortex (Rizzolatti et al., 1981a,b). Multisensory neurons are able to respond to different combinations of sensory inputs−visual, tactile, proprioceptive, vestibular, auditory−when these inputs are perceived simultaneously, for example when a visual input (e.g., a looming cue) is perceived at the same time as a tactile stimulus (e.g., a tap on the hand). The functioning of these multimodal neurons is comparable to the multisensory integration occurring within the superior colliculus, where convergent visual, auditory, and somatosensory stimuli elicit maximal neuronal activity (Meredith and Stein, 1986; Cardinali et al., 2009). Furthermore, the evoked response in multimodal neurons is mediated by the distance between the visual object and the tactile receptive field (RF), such that the appraisal of visual information is dependent on how close this visual input is to the body part containing the tactile receptive field (Brozzoli et al., 2012b). Multisensory neurons, dedicated to the representation of PPS, have been identified in different brain regions of macaque monkeys, and specifically within the premotor and parietal cortex, and the putamen (Rizzolatti et al., 1997; Fogassi et al., 1999; Graziano, 2001; Graziano and Cooke, 2006).

The ventral-caudal premotor cortex (also called area F4), adjacent to the ventral-rostral premotor cortex (area F5) and to the primary motor cortex, contains multisensory neurons that respond to both visual and tactile stimuli, and a somatotopic map of arms, hands, and face (Holmes and Spence, 2004). Interestingly, electrical stimulation of area F4 in monkeys (labeled as a polysensory zone due to the neuronal response to multisensory stimuli; Graziano and Cooke, 2006) results in a defensive movement aimed at protecting the body (withdrawing and blocking movements). Within the posterior parietal cortex, the ventral intraparietal sulcus, Brodmann area 7, and Brodmann area 5 contain multimodal neurons that have been associated with PPS in monkeys (Holmes and Spence, 2004; Brozzoli et al., 2012b). Here, neurons shape a gross somatotopic representation (reproducing the same arrangement as the body surface such that neighborhood relation are preserved), with tactile RFs dedicated to the face, arm, and head. Of note, the ventral intraparietal sulcus is in close proximity to the medial intraparietal sulcus, also called the parietal reach region due to its role in reaching action representation (Koob et al., 2010). While the ventral and medial intraparietal areas are known to be interconnected and functionally intertwined (Grefkes and Fink, 2005), they are considered as pertaining two distinct neural networks (one for PPS and one for reaching representations, respectively). Other brain areas involved in PPS representation in monkeys include the parietal occipital junction (Brozzoli et al., 2012b) and the putamen (Graziano and Gross, 1994). Whereas the parietal occipital junction is thought to carry a face- and hand-centered representation of visual space, the putamen appears relevant to visuo-tactile processing of the space around the body and contains visuo-tactile neurons with tactile RFs on the arm, hand, and face that are somatotopically organized.

Another notable characteristic observed through neurophysiological studies of PPS in monkeys is the observation that neurons in the premotor cortex activated by the presence of an object in the space surrounding the body continue firing in the absence of the visual stimulus if the monkey believes the stimulus is still there, thus suggesting that PPS is maintained internally on the basis of previous experience (Graziano et al., 1997a).

### Neural Correlates of Peripersonal Space in Humans

Studies examining the neural correlates of PPS in humans reproduce largely findings in monkeys. Emerging work using electroencephalography to record electrical brain activity in humans (Sambo and Forster, 2011; Bernasconi et al., 2018; Noel et al., 2019) showed electrodes corresponding to somatosensory-motor regions in the parietal cortex to be associated with PPS representation. In particular, the study conducted by Bernasconi et al. (2018), using intracranial electroencephalography, was able to identify specific cortical and subcortical regions involved in multisensory integration and PPS representation. Here, they demonstrated that the postcentral gyrus, insula, and parahippocampal gyrus are central to the encoding of spatio-temporal aspects of PPS. While the postcentral gyrus has been well established as brain region underlying PPS, the insula and parahippocampal gyrus appear to be novel in the conceptualization of PPS representation.

**FIGURE 1:**
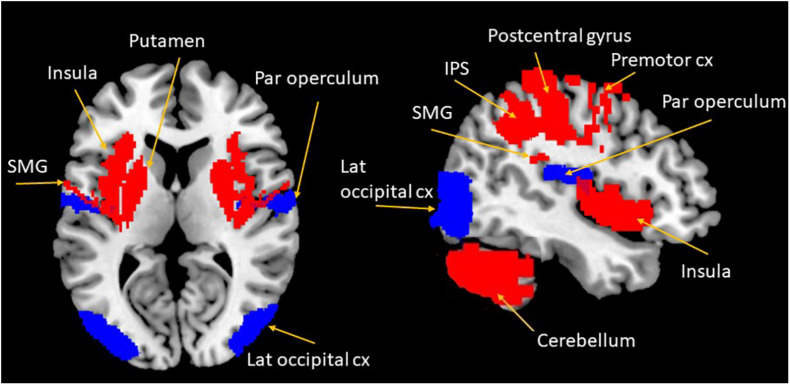
Overview of cortical and subcortical regions involved in the PPS representation and in PTSD. Blue areas show regions primarily involved in the PPS representation; red areas show regions associated with both PPS representation and PTSD. Cx, cortex; IPS, intraparietal sulcus; lat, lateral; SMG, supra marginal gyrus; par, parietal.

A wealth of functional neuroimaging studies have further established a PPS neural network that encompasses the ventral intraparietal sulcus, the lateral occipital cortex, and the ventral premotor cortex (see Figure 1; Makin et al., 2007; di Pellegrino and Làdavas, 2015). The ventral intraparietal (VIP) sulcus has been associated with the visual guidance of head movements, as well as hand, eye, and mouth coordination, further eliciting complex defensive movements (eye closure, facial grimacing, and protective movement of the hand were elicited via electrical stimulation of the VIP in monkeys; Cooke and Graziano, 2003) under perceived threat. Most VIP neurons integrate visual and tactile afferent information, with a matching representation of body surface appearing present within this area (Squire, 2009). Similarly to the primate brain, the VIP in humans is specifically associated with PPS representation, despite its proximity and interconnections with other distinct intraparietal areas associated with visual-motor coordination for reaching and grasping (e.g., anterior and medial intraparietal areas; Grefkes and Fink, 2005). The lateral occipital complex (extra-striate regions in the human visual cortex) is also involved in shape perception and object recognition (Grill-Spector et al., 2001) and is essential to representation of the surroundings of the body.

Conversely, the ventral premotor cortex is involved in hand movements, recognition of other individuals’ hand movements, motor aspect of speech, associative sensorimotor learning and sensorimotor integration (Binkofski and Buccino, 2006). For example, one study revealed a close resemblance between monkey and human brains relative to multimodal processing of moving stimuli in the intraparietal sulcus (equivalent to the ventral intraparietal area in monkeys), the ventral premotor, and the lateral postcentral cortex−the site of the primary somatosensory cortex (Bremmer et al., 2001). More recent studies investigating the integration of visuo-tactile stimuli in the human brain find that the intraparietal sulcus, supramarginal gyrus, parietal operculum (second somatosensory area), insula, dorsal premotor cortex, cerebellum (lobule VII), and putamen emerge as key areas containing multimodal neurons that integrate visuo-tactile stimuli in the near-hand space (Gentile et al., 2011, 2013). Critically, all these brain regions have shown activation during visuo-tactile stimulation of the hand in humans (where the tactile stimulus was delivered on the hand and the visual stimulus was displayed at ∼2 cm above the hand; Gentile et al., 2011) and are involved in visuo-spatial and visuo-motor functions essential to the multisensory representation of the space surrounding the body (Gentile et al., 2011). Interestingly, a study by the same research group found the ventral premotor area to serve as the focus of whole body-centered multimodal processing (Gentile et al., 2015). In addition, the superior parietal occipital junction appears to play a critical role in representing a hand- and face-centered visual space surrounding the body (Gallivan et al., 2011). Finally, recent research reveals the role of the vestibular system in modulating PPS boundaries. Here, as compared to no rotation, vestibular stimulation (whole-body rotation) that was congruent with the direction of external inputs (leftward or rightward approaching auditory stimuli) was found to speed tactile detection and expand PPS boundaries around the body (as measured by tactile response time; Pfeiffer et al., 2018).

Crucially, the neural correlates underlying PPS representation appear to overlap with brain areas associated with other aspects of bodily self-consciousness. For example, fronto-parietal regions that process multisensory information are linked to both PPS and body ownership (Blanke, 2012; Serino, 2019). In addition, the insula and the putamen have been linked to both PPS and body ownership tasks (Blanke, 2012). Of note, a conjunction analysis by Grivaz et al. (2017) identified only two small activation clusters within the left parietal cortex during tasks involving PPS and body ownership (Grivaz et al., 2017; Serino, 2019). Furthermore, the posterior parietal cortex and vestibular regions associated with PPS representation have also been found to be involved in processing self-location and first-person perspective (Blanke, 2012). Taken together, neuroimaging data support the hypothesis of interconnection and functional interaction among different aspects of bodily self-consciousness necessary to create a coherent conscious experience; however, further studies are warranted to better understand the neural correlates underlying different aspects of bodily self-consciousness.

## Peripersonal Space and Psychological Traits and Disorders

As illustrated in Table 1, consistent with ongoing conceptualizations of the PPS as a protective zone surrounding one’s body (a so-called defensive peripersonal space), specific personality traits and psychopathologies have been found to affect the PPS representation and its plasticity. We conducted a literature review using PubMed and the following search keywords: “personal space” in addition to “anxiety,” “autism,” “schizophrenia,” “PTSD,” “disorder,” respectively. The rationale behind the keywords stemmed from previous PPS research reporting specific PPS characteristics in anxiety, autism, and schizophrenia (Noel et al., 2015b, 2018b), and from our interest in PTSD in particular. The keyword ‘disorder’ was used to explore potential additional research in psychopathology. The studies included used evidence-based methodology to measure PPS boundaries, a valid standardized psychological measure for assessment, and involved adults. The present literature search was intended to provide a background of the research on PPS in psychopathology, and did not intend to cover the entire literature on psychological traits and PPS in a systematic fashion. Taken together, the studies presented point toward a significant correlation between PPS representation and specific psychological characteristics. Further studies are warranted to clarify whether specific characteristics of the PPS underlie the etiology of psychological disorders and how psychological states and traits can affect the representation of an individual’s surrounding space (see Table 1 for a summary).

**TABLE 1:** List of studies investigating PPS in psychopathology.

References	PPS measure	Sample	Psychological characteristics	Psychological assessment	Results
Bogovic et al., 2014	The variable measured is Personal Space (PS), through a stop-distance paradigm, where participants self-judge PS boundaries and communicate them verbally with respect to a person approaching them	83 male veterans with PTSD, 68 male veterans without PTSD	PTSD	PTSD prior diagnosis according to DSM IV, Mississippi Scale for Combat-Related Posttraumatic Stress Disorder (M-PTSD)	Participants with PTSD showed a significant preference for greater interpersonal distances as compared to the control group. The PTSD group also showed a preference for greatest interpersonal distance when approached from behind. By contrast, the control group preferred the greatest distance when approached frontally.
Delevoye-Turrell et al., 2011	The variable measured is Personal Space (PS), through a stop-distance paradigm, where participants self-judge PS boundaries and communicate them verbally with respect to either an object or a person.	20 individuals with schizophrenia (SCZ), 20 paired-matched healthy controls (HC)	Schizophrenia	Positive and Negative Syndrome Scale-PANSS for individuals with Schizophrenia	Either in the object condition and the person condition, SCZ participants revealed increased judgment variability of PS boundaries, as compared to HC.
Di Cosmo et al., 2018	Time reaction to a tactile stimulus administered on the hand, while task-irrelevant approaching sounds are presented	Study 1: 20 adults with Schizophrenia (SCZ), 20 healthy participants (HC). Study 2: out of 36 healthy participants, 18 were included in the low-schizotypy group and 18 in the high-schizotypy group	Study (1) Schizophrenia Study (2) Schizotypal traits	Positive and Negative Syndrome Scale-PANSS for individuals with Schizophrenia, Schizotypal Personality Questionnaire-SPQ for healthy controls	Study 1: SCZ showed significantly narrower and sharper PPS boundaries as compared to HC, as measured through independent sample t-test on the reaction time to tactile hand stimulation during approaching sounds. Study 2: independent sample t-test showed high-schizotypy group had significantly narrower PPS boundaries than the low-schizotypy group
Hunley et al., 2017	Line bisection bias in the far and near space with a laser point or a stick	70 healthy participants	Claustrophobic fear	CLQ Claustrophobia Questionnaire	In the laser point condition, the suffocation subscale scores of the CLQ were found to be significant independent predictors of the size of near space (PPS), using multiple least-squares regression, where higher scores corresponded to larger PPS. In the stick condition, individuals with higher claustrophobic fear did not show expansion of PPS when bisecting lines at farther distances.
Iachini et al., 2015	variable measured is Personal Space (PS), through a stop-distance paradigm, where participants self-judge PS boundaries and communicate them verbally with respect to a person approaching them	70 healthy participants	Trait anxiety	STAI State-Trait Anxiety Inventory	The correlation analyses showed that reachability space and comfort-distance judgments were positively correlated to both trait and state anxiety scores.
Lourenco et al., 2011	Line bisection bias in the far and near space	35 healthy participants	Claustrophobic fear	CLQ Claustrophobia Questionnaire	CLQ scores were found to be significant independent predictors of the size of near space (PPS), using multiple least-squares regression, where higher scores corresponded to larger PPS.
Mul et al., 2019	Time reaction to a tactile stimulus administered on the hand, while task-irrelevant approaching sounds are presented	22 adults with autism spectrum disorders and 29 healthy participants	Autism Spectrum Disorder	Autism Diagnostic Observation Schedule diagnostic interview (ADOS)	ASD participants exhibited a smaller PPS size as compared to healthy controls, and showed to have sharper PPS boundaries, as measured by the slope in their RTs as a function of the temporal delay of the tap, when they were approached by sound.
Sambo and Iannetti, 2013	Hand-blink reflex	15 healthy participants	Trait anxiety	STAI State-Trait Anxiety Inventory	Using multiple least-squares regression, trait anxiety resulted to be a significant predictor of PPS size, where higher anxiety scores corresponded to a larger PPS size.
Taffou and Viaud-Delmon, 2014	Audio-tactile interaction task: time reaction to vibration stimulation of a finger, during presentation of threatening vs. non-threatening approaching sounds.	30 healthy participants: 15 with low cynophobia scores, 15 with high cynophobia scores	cynophobia-unreasonable fear of dogs	Questionnaires from the authors exploring fear of dogs	Participants with high cynophobic scores showed larger PPS boundaries as compared to participants with low cynophobic scores during the threatening sound condition (growling dog).

### Trait Anxiety

As described above, the hand-blink reflex has been used to measure the defensive response within the PPS. This brainstem-driven reflex is modulated by the sensorimotor cortices responsible for representation of PPS (Sambo et al., 2012). Here, Sambo and colleagues investigated the correlation between the hand-blink reflex and trait anxiety in a student sample. They found a significant positive correlation between the size of the PPS and trait anxiety, suggesting that the higher the level of anxiety experienced by an individual, the larger the PPS as indexed by the magnitude of the hand-blink reflex (Sambo and Iannetti, 2013). Interestingly, however, no such correlation emerged from claustrophobic traits in this study (see below). A following experiment showed similar results, where trait anxiety was found to be positively correlated with a larger peripersonal space in a healthy sample (Iachini et al., 2015).

### Phobias

Using a line bisection task in far and near space (where a leftward versus rightward bias is usually employed to differentiate between the near versus far space, respectively), Lourenco and colleagues (Lourenco et al., 2011) found that individuals reporting higher claustrophobic scores had a larger PPS (i.e., they showed a leftward bias within a larger space around the body). These authors suggested that a larger PPS may be critical to the etiology of claustrophobia. Critically, similar results were reported in a more recent study where subjects endorsing high levels of claustrophobic fear had a larger PPS when compared to those with low claustrophobic fear while using a laser point to perform the line-bisection task (Hunley et al., 2017). These researchers also found that people with high claustrophobia scores have a decreased ability to expand the PPS when using a tool (a stick) to perform the line bisection task, thus showing decreased flexibility of the PPS. Another study examining cynophobia (unreasonable fear for dogs) (Taffou and Viaud-Delmon, 2014), using an audio-tactile interaction task, found that a group of dog-fearful participants showed a larger PPS as compared to a group of non-dog-fearful participants when exposed to a threatening growling dog sound.

### Autism

A recent study found that adults with autism spectrum disorder had a PPS that was smaller and with sharper boundaries (steeper slope in reaction times between extrapersonal and peripersonal space) than those observed in healthy controls (Mul et al., 2019). Here, the researchers employed a standard audiotactile task (tactile stimulation on a finger while an irrelevant looming sound was delivered) and used reaction times as an indirect measure of PPS.

### Schizophrenia

A recent study investigated PPS in schizophrenic adults (Di Cosmo et al., 2018) using a standard audio-tactile task (where reaction times to a tactile stimulus to the hand were used as a proxy of PPS boundaries, while irrelevant looming sounds were administered to the participant). Here, a smaller PPS seemed to characterize individuals with schizophrenia as compared to healthy controls. Additionally, individuals with higher scores on the schizotypal traits, as measured through the Schizotypal Personality Questionnaire (Raine, 1991), showed smaller PPS as compared to individuals with lower schizotypal traits. This result was interpreted as evidence of the abnormal representation of the space surrounding the body in schizophrenia. In line with this conclusion, a study using self-judgment as a measure of PPS boundaries (participants indicated verbally when they thought a target−person vs. object−reached the boundaries of their PPS) found that as compared to healthy controls, participants with schizophrenia showed high variability in defining their PPS through self-judgment (Delevoye-Turrell et al., 2011). This result is in keeping with the conceptualization that schizophrenia is a disorder of the self, where PPS boundaries are not clearly defined and/or a high variability characterizes them, depending on the symptomatology present moment by moment (Noel et al., 2017).

## How Trauma May Affect Peripersonal Space

### Peripersonal Space in Trauma-Related Disorders

Experiencing trauma has the potential to strongly impact the individual’s life, with associated biological, social, and psychological repercussions. In trauma-related disorders (PTSD, Acute Stress Disorder, Adjustment Disorder) hyper-arousal, re-experiencing, avoidance, negative mood and cognition, as well as dissociative symptoms may occur, with affected individuals enduring significant impairment in their lives (APA, 2013). This array of symptoms can alter the representation of the self at multiple levels, including at the cognitive, bodily, and social levels, thus leading to significant effects on how an individual interacts with her/his environment. Here, we explore alterations of bodily self-consciousness as they relate to symptoms of depersonalization and of derealization, where an individual feels detached from his/her entire body or parts of the body, or when surroundings are perceived as unreal or dream-like, respectively (Lanius et al., 2010; APA, 2013; Stein et al., 2013).

Critically, altered neural activity and connectivity associated with PTSD symptomatology shows significant overlap with cortical and subcortical regions that have been found to be involved in the PPS representation (e.g., Lanius et al., 2006, 2016; Shaw et al., 2009; Shin and Liberzon, 2010; Daniels et al., 2012; Whalley et al., 2013; Teicher et al., 2016; Wang et al., 2016; Naegeli et al., 2018).

**FIGURE 2:**
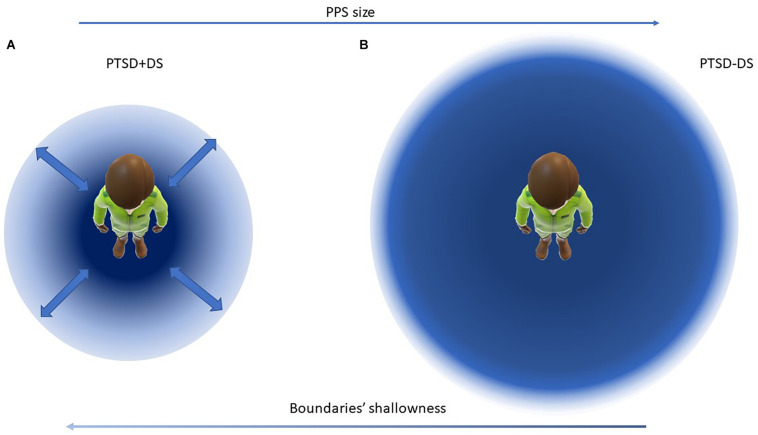
Visualization of the proposed model of PPS representation in individuals with the dissociative subtype of PTSD **(A)** and with PTSD **(B)**. In panel **(A)** the PPS is smaller and with shallow boundaries, in panel **(B)** the PPS is larger and with sharper boundaries. Arrows indicate progressive increase of sharp shift peri-extra-personal space toward the right, and increase of shallowness of PPS boundaries toward the left. Bold arrows indicate high variability in the PPS size in panel **(A)**.

Accordingly, we propose that trauma-related symptoms may affect the representation of PPS, especially when considering its defensive purpose. As previous research demonstrates, psychological traits can influence PPS boundaries: whereas, for example, trait anxiety and claustrophobia have been correlated with larger PPS boundaries (Lourenco et al., 2011; Sambo and Iannetti, 2013; Hunley et al., 2017), schizotypal traits and autism have been linked to smaller and sharper PPS boundaries (Di Cosmo et al., 2018; Mul et al., 2019). To date, however, no research has explored specifically the influence of trauma exposure on the PPS representation. Considering that hyper-arousal symptoms involve a heightened alertness and hyper-vigilance (APA, 2013), we hypothesize that individuals diagnosed with PTSD and Acute Stress Disorder will exhibit a larger PPS as compared to controls as a self-protective function, thus ensuring a bigger safety zone surrounding their body. In addition, avoidance symptoms−the tendency to fleeing from trauma reminders in the form of places, interactions, emotions (APA, 2013)−are likely to require larger PPS boundaries in order to avoid interpersonal contact. Consistent with this hypothesis, one study investigating interpersonal space representation in a population of male veterans (Bogovic et al., 2014) found that when asked to state their preferred interpersonal distances, participants with combat-related PTSD preferred significantly larger interpersonal distance as compared to controls. Interestingly, greater interpersonal distance was also preferred by PTSD participants when the other person was approaching the back of the participant as compared to the front. Finally, in a previous study using the Rubber Hand Illusion experimental paradigm, as compared to healthy controls, we observed a more rigid representation of the body among individuals with PTSD (Rabellino et al., 2018a). As pointed out by previous literature reviews (Ehrsson, 2012; Serino, 2019), the interdependence among different aspects of bodily self-consciousness allows us to unravel characteristics of the PPS representation while observing the expression of self-identification (e.g., body ownership). Here, individuals with PTSD were significantly less affected by the illusion effect of owning a fake hand instead of their own (as revealed by a smaller perception drift), thus showing a less malleable representation of the bodily self. Taken together, these observations suggest that in PTSD the PPS is likely to be characterized by a larger size (for defensive purposes) and limited plasticity and dynamics (less susceptibility to modify the body schema in adaptation to changes in multisensory inputs, which would in turn lead to a rigid PPS; see Figure 2). In addition, we would expect to observe sharp PPS boundaries in PTSD−including a sharp contrast in response to stimuli in the near versus far space−, similar to a previous study that investigated PPS in association with trait anxiety (Sambo and Iannetti, 2013).

By contrast, the dissociative subtype of PTSD, characterized by depersonalization and derealization (APA, 2013) is likely to have the effect of preventing the individual from creating a stable space as a defensive zone around the body. During states of depersonalization involving detachment from one’s own body, the individual lacks the ability to maintain a coherent representation of the body (Lanius et al., 2012; Spiegel et al., 2013; Ataria et al., 2015). When this is lost, one may speculate that the PPS might become highly variable in size, depending on the psychological state of the individual (for example, changing from an embodied state to a state of depersonalization). Indeed, in our own study of sense of ownership in PTSD and its dissociative subtype (Rabellino et al., 2016b, 2018a), we observed high variance in the rubber hand illusion effect among individuals within the dissociative subtype group. Here, some of participants switched from a very rigid representation of the body (very small illusion effect, as measured by the proprioceptive drift) to a high susceptibility to the illusion during the experiment (as measured by an increased proprioceptive drift and an endorsement of the subjective illusion effect). We suspect that significant disruption of multisensory integration processes, among individuals with dissociative symptoms, manifested as either a hyper-rigid (as in non-dissociative PTSD) or extremely weak representation of the body during characterization of the flow of experience (as experienced in out-of-body experiences, where the individual perceives him/herself as out of the body; Ataria, 2016). We thus expect the emergence of extremely variable, state-dependent PPS boundaries among individuals with the dissociative subtype of PTSD, where the boundaries of the PPS representation would vary depending on the psychological state of the individual (for example, transitioning in and out of a state of depersonalization; see Figure 2).

Similarly, a review of studies on peripersonal space in schizophrenia and in autism spectrum disorder (see also above paragraph “Peripersonal Space and Psychological Traits and Disorders;” Noel et al., 2017) indicated that individuals diagnosed with schizophrenia experience shallow and highly variable boundaries between the peri- and extrapersonal space (as measured via various body-illusion experiments). Shallow and highly variable PPS boundaries were thus interpreted as an impairment in distinguishing between the self and others, a difficulty in keeping with the aforementioned conceptualization of schizophrenia as a disorder of the self (Noel et al., 2017). Accordingly, we hypothesize that disorders of consciousness, including depersonalization disorders, are likely to involve similar shallow boundaries surrounding PPS representation, with high variability characterizing the delimitation of the PPS. This high variability in PPS boundaries could correspond to an unstable self-other distinction in the moment-to-moment flow of bodily self-consciousness. In fact, the dissociative subtype of PTSD often presents a comorbidity with other dissociative disorders (e.g., dissociative amnesia, dissociative identity disorder; APA, 2013; Wabnitz et al., 2013; Swart et al., 2020) and with associated instability of the sense of self.

Sense of agency − the sense of controlling one’s own body actions (De Vignemont and Fourneret, 2004; Haggard and Chambon, 2012) is another intriguing aspect of self-consciousness. In a recent study on the relation between sense of agency, body schema, and PPS (D’Angelo et al., 2018), sense of agency was found to shape body schema and PPS, where an increased sense of agency appeared to extend the boundaries of the PPS. Experiencing trauma also profoundly affects the sense of agency (Ataria, 2015) as the traumatic event itself can produce a loss in the sense of agency, at least temporarily. For example, during a traumatic event, an individual may experience the body as not responsive to one’s intentions, an experience defined as a freezing/tonic immobility state, where the body feels frozen and unable to move (Kozlowska et al., 2015; Volchan et al., 2017) or, alternatively, may experience extreme helplessness when the event itself involves the impossibility of action (e.g., when no physical escape is viable; Schauer and Elbert, 2010). Indeed, our study using the rubber hand illusion paradigm to investigate sense of ownership and agency in PTSD revealed a negative correlation between the illusion effect and sense of agency in individuals with PTSD, where a higher illusion effect corresponded to a decreased sense of agency (Rabellino et al., 2018a). Taken together, these studies suggest that individuals with PTSD who report a decreased sense of agency, especially the dissociative subtype of PTSD, would show unique PPS representations.

### Neural Correlates of Peripersonal Space in Trauma-Related Disorders

In this section, we aim to present the interesting overlap between the neural substrates underlying PTSD and the neural correlates involved in the PPS representation. A range of neuroimaging studies involving individuals with PTSD provides strong evidence for altered neural activity and connectivity in cortical and subcortical brain structures that are also implicated in key processes underlying the representation of PPS, including multisensory processing, bodily perception, and self-consciousness (see Figure 1). The following section explores how altered neural activity in PTSD in key brain regions involved in PPS may influence PPS in this disorder.

#### Premotor Cortex

As compared to healthy controls, victims of urban violence with PTSD show reduced volume in the ventral portion of the premotor cortex (Rocha-Rego et al., 2012), a region central to sensorimotor integration, hand movements, and the motor aspect of speech (Binkofski and Buccino, 2006). By contrast, the dorsal part of the premotor cortex, associated with the multisensory representation of the PPS, shows increased activity during reliving symptoms in PTSD (Whalley et al., 2013) and hyper-responsiveness to alerting sounds in PTSD as compared to trauma-exposed controls (Naegeli et al., 2018). Functional connectivity studies in PTSD reveal further increased connectivity of the premotor cortex with the dorsolateral periacqueductal gray, a brainstem structure involved in active defensive responses (such as fight/flight responses), thereby suggesting increased active defense responses in PTSD at rest (Harricharan et al., 2016). In addition, weakened connectivity between the premotor cortex and middle frontal gyrus has been shown in individuals with PTSD during implicit threat processing, a finding in keeping with the widespread documentation of disruption in executive functioning among individuals with PTSD (Cisler et al., 2013). Taken together, these studies point toward altered neural activity and connectivity of the premotor cortex in PTSD, suggesting a trauma-related differential functionality of the sensorimotor integration in this disorder, likely affecting the representation of PPS.

#### Intraparietal Sulcus

The intraparietal sulcus, a region fundamental to PPS representation and also containing multimodal neurons for multisensory processing, appears hyper-responsive to exposure to loud sounds among individuals with PTSD as compared to trauma-exposed controls (Naegeli et al., 2018). This result suggests atypical noradrenergic influences stemming from the locus coeruleus, an effect likely to trigger attention and motor preparation processes associated with an alerting state, thus affecting sensorimotor processing and PPS representation in PTSD.

#### Putamen

In addition to its well-known role in motor planning and in executive functioning (Gentile et al., 2011), the putamen is also involved in visuo-tactile integration. For example, one study examining PTSD in relation to interpersonal trauma reported increased functional connectivity of the locus coeruleus with the putamen in PTSD (Steuwe et al., 2015). The putamen has also shown to be hyper-active among individuals with PTSD in association with reliving symptoms (Osuch et al., 2001), and at rest (Wang et al., 2016) and has shown to be positively correlated with PTSD symptom severity during trauma processing (Mickleborough et al., 2011) and autobiographical memory retrieval (Thome et al., 2020). Taken together, these findings point toward the important role played by the putamen in the planning of motor actions in the surrounding environment, thus likely affecting the goal-oriented function of PPS.

#### Supramarginal Gyrus

The supramarginal gyrus is a crucial area for bodily self-consciousness and for maintaining a stable representation of one’s body in space (self-location; Serino et al., 2013; Blanke et al., 2015). One study investigating neural activity in PTSD found increased activity of the supramarginal gyrus during flashbacks − a common symptoms in PTSD where the individual re-experiences a traumatic event as if reoccurring in the present moment − (Whalley et al., 2013), a time when bodily self-consciousness and self-location are likely disrupted. By contrast, a metanalysis of neuroimaging studies of PTSD reported that the supramarginal gyrus is among those neural areas showing decreased neural activity when compared to controls (Patel et al., 2012). Furthermore, in our own work examining the functional connectivity of two areas of the cerebellum involved in sensorimotor integration (the anterior cerebellum and the anterior vermis) in PTSD, we found decreased functional connectivity of these regions with the supramarginal gyrus among individuals with the dissociative subtype of PTSD as compared to PTSD and healthy controls (Rabellino et al., 2018b). These results are suggestive of an impairment in maintaining a stable sense of bodily self-consciousness in the dissociative subtype of PTSD. Similarly, a study investigating the functional connectivity of the vestibular nuclei (involved in bodily orientation in space) in PTSD at rest found that the functional connectivity of the vestibular nuclei with the supramarginal gyrus was negatively correlated with depersonalization/derealization symptoms scores (Harricharan et al., 2017). This result pointed further toward an impaired sense of bodily orientation in space among individuals experiencing depersonalization states, a finding consistent with the results of previous studies suggesting altered functioning of the supramarginal gyrus during induced out-of-body experiences (De Ridder et al., 2008; Lopez et al., 2008). Taken together, these findings suggest that altered bodily self-consciousness, associated strongly with the dissociative subtype of PTSD, may affect functionality of the PPS representation in this disorder.

#### Postcentral Gyrus

Notably, the postcentral gyrus, also involved in motor responses and PPS representation, shows increased neural activity, as compared to healthy controls, among individuals with PTSD during attention and working memory tasks (Brown and Morey, 2012). By contrast, decreased activation of the bilateral postcentral gyri was reported in a meta-analysis investigating neural activity during symptom provocation (trauma-related stimuli) among individuals with PTSD as compared to controls (Sartory et al., 2013). Moreover, decreased functional connectivity of the postcentral gyrus with the anterior cerebellum (lobule IV-V) and the anterior vermis (cerebellar regions involved in sensorimotor processing and integration) has been reported in the dissociative subtype of PTSD when compared to PTSD and healthy controls (Rabellino et al., 2018b), thus supporting our hypothesis of impaired functionality of sensorimotor processing and integration in the dissociative subtype of PTSD. Finally, decreased functional connectivity between the insula and postcentral gyrus has been reported in PTSD and its dissociative subtype as compared to controls, pointing toward impairment in the monitoring and emotional appraisal of the surrounding environment (Harricharan et al., 2020). On balance, these results support further the presence of alterations of sensorimotor processing and multisensory integration in PTSD, an effect particularly pronounced in the dissociative subtype of PTSD, which may be associated with altered representation of PPS.

#### Cerebellum

Different regions of the cerebellum (especially anterior cerebellum and anterior vermis that are of interest for bodily self-consciousness and PPS representation) have shown altered neural activity and functional connectivity in PTSD and its dissociative subtype either during trauma processing and at rest (Bonne et al., 2003; Yin et al., 2011; Wang et al., 2016; Carletto and Borsato, 2017). In particular, neural activity within the anterior cerebellum (lobules IV-V) was found to correlate positively with flashback intensity in PTSD (Osuch et al., 2001). In addition, increased regional cerebral blood flow (rCBF) has been reported within the anterior vermis among individuals with combat-related PTSD during exposure to combat sounds as compared to neutral sounds (Pissiota et al., 2002). At rest, increased resting-state functional connectivity of the anterior vermis with the amygdala − involved in emotional response − and periaqueductal gray − a key region for the fight-or-flight response under threat − (Thome et al., 2016) was demonstrated in PTSD as compared to healthy controls. Furthermore, decreased functional connectivity of the anterior cerebellum and anterior vermis with temporo-parietal regions and somatosensory cortices (postcentral and supramarginal gyrus), brain regions essential to multisensory integration and bodily self-consciousness, has been reported in the dissociative subtype of PTSD as compared to PTSD and healthy controls at rest (Rabellino et al., 2018b). By contrast, in the same report, increased functional connectivity of the posterior cerebellum (Crus I, involved in cognitive functioning) with multisensory processing cortices (temporal pole) was observed in the dissociative subtype of PTSD as compared to PTSD at rest (Rabellino et al., 2018b). Finally, reduced cerebellar volume has been reported in PTSD (De Bellis and Kuchibhatla, 2006; Carrion et al., 2009; Baldaçara et al., 2011), a finding suggestive of decreased functioning of bodily self-consciousness processes. Collectively, these observations of alterations in the neural activity and functional connectivity of cerebellar regions essential to sensorimotor integration are in line with an overall pattern of impaired functioning of bodily self-consciousness in PTSD, which may in turn have consequences on the processing and integration of inputs representing the surrounding environment.

#### Insula

The insula serves as a fundamental structure for interoceptive awareness. Interoception plays a crucial role in bodily self-consciousness and represents an essential function for differentiating internal and external sensory inputs. Interestingly, PTSD is often characterized by hyperarousal symptoms, where interoception (internal signals such as heart rate, breathing rate, sweating) may overload the conscious experience of the bodily perception. By contrast, the dissociative subtype of PTSD is characterized by detachment from the body, including bodily signals, with a resulting impairment of interoceptive awareness (Harricharan et al., 2020). In numerous neuroimaging studies of individuals with PTSD, the insula appears hyper-responsive during various tasks involving trauma processing or presentation of emotional stimuli (Lanius et al., 2007; Simmons et al., 2008; Shin and Liberzon, 2010; Aupperle et al., 2012; Brinkmann et al., 2017) and its neural activity correlates positively with symptom severity (Osuch et al., 2001; Hopper et al., 2007). In addition, functional connectivity studies have revealed increased functional connectivity between the insula and amygdala − involved in emotional responsiveness − (Nicholson et al., 2016) in PTSD, and between insula and BNST − associated with sustained threat response − in the dissociative subtype of PTSD (Rabellino et al., 2017) as compared to healthy controls, suggesting an association between interoceptive awareness and fear response.

A more recent study examining the functional connectivity of the insula in PTSD at rest showed decreased functional connectivity of the insula with sensorimotor cortices (pre- and post-central gyri) in PTSD as compared to healthy controls, with even weaker connectivity observed in the dissociative subtype as compared to the PTSD group. These findings have been discussed in relation to the limited capacity of individuals with PTSD and its dissociative subtype to integrate afferent information from internal states and from the environment into a unified conscious experience (Harricharan et al., 2020), an ability fundamental to maintain an integrated bodily self-consciousness and create an effective representation of the PPS.

#### Vestibular System

Finally, the vestibular system serves as fundamental neural network involved in interoceptive awareness and multisensory information processing. This latter is essential to orient the body in space and has been considered crucial to the PPS representation. Vestibular neurons have been indicated to be involved in trimodal visuo-tactile vestibular integration to allow the processing of self-location and self-identification, two main aspects of bodily self-consciousness (Blanke, 2012). Interestingly, a recent study investigating the functional connectivity of the vestibular nuclei in the brainstem of individuals with PTSD at rest (Harricharan et al., 2017) showed decreased connectivity of the vestibular nuclei with the posterior insula in PTSD as compared to controls, and disrupted connectivity with key cortical vestibular areas (parieto-insular and dorsolateral prefrontal cortex) in the dissociative subtype of PTSD as compared to PTSD and controls. These data suggest a disturbed body orientation in space in PTSD that would affect the representation of the body and related PPS. Previous studies in neurological patients have described heautoscopic symptoms (visual illusion of seeing one’s body from out of the body, often from above) as self-identification and self-location disorders, where impaired multisensory integration of bodily signals and disintegration of vestibular signals may be at the root of out-of-body experiences (Blanke and Mohr, 2005; Blanke, 2012). Here, an interesting research question arises about the nature of the PPS representation during dissociative experiences associated with psychological trauma-related disorders (dissociative subtype of PTSD; Frewen et al., 2015). Importantly, we argue that state-dependent dissociative symptoms (affected by the psychological temporary state; Frewen and Lanius, 2014; Lanius, 2015) would facilitate the investigation of temporary dynamic distortions of the PPS, thus deepening our understanding of bodily self-consciousness as a dynamic experience that changes over time.

Taken together, the neuroimaging studies reviewed here support the hypothesis of an atypical representation of the PPS in PTSD, where neural structures related to bodily self-consciousness, self-location, motor planning, and multisensory integration show altered neural activity and/or functional connectivity in PTSD and its dissociative subtype when compared to controls. This neural pattern can be interpreted as a general reorganization of neural networks in PTSD that favors innate alarm responses to potential threats and preparation for defense responses, thus affecting the integration of multisensory afferent and efferent inputs, with the resulting representation of the bodily self and its surroundings being biased toward potential incoming threats (Rabellino et al., 2015, 2016a, 2019; Steuwe et al., 2015; Lanius et al., 2016; Frewen et al., 2017).

## Conclusion

On balance, our review of PPS and its relation to trauma-related disorders points clearly to the need for further study within this field and, overall, aims to inspire future directions of research on PPS and bodily self-consciousness in the aftermath of trauma.

Although this work provides an extensive overview of PPS characteristics and its relationship with psychological traits and symptoms, it is not intended as a systematic review but rather as a perspective contribution to the understanding of PPS and trauma-related disorder. Investigation of the different aspects of PPS representation (function, plasticity and dynamics, neural correlates) in psychopathology can deepen our scientific and clinical understanding of bodily self-consciousness, particularly in relation to the altered states of consciousness that underlie dissociative symptoms characterizing certain psychological disorders, including trauma-related disorders. Depersonalization and derealization symptoms remain among the most difficult psychological symptoms to recognize, diagnose, and treat, despite seriously impacting the quality of life of individuals (Boyd et al., 2018). A greater understanding of the PPS representation in trauma-related disorders has the potential to lead to innovative diagnostic methods for recognizing dissociative symptoms, as well as the development of novel clinical interventions designed to specifically target these symptoms. In addition, enhanced knowledge of bodily self-consciousness in relation to the immediate surroundings of an individual may give rise to targeted treatment approaches that could improve multisensory integration in potentially stressful situations, such as social contexts, thus improving social relationships among individuals with trauma-related disorders.

Given that trauma-related disorders are also associated with impairment of bodily self-consciousness in relation to a threat response (Kozlowska et al., 2015; Lanius, 2015; Ataria, 2016), investigation of PPS in this population has the potential to elucidate further the defensive purpose of PPS, while clarifying its role in innate alarm responses under threat. Indeed, preliminary studies examining the flexibility of body schema in PTSD and its dissociative subtype (Rabellino et al., 2016b, 2018a) appear promising in pointing toward the need for further research on the plasticity and dynamics of PPS, which together have the potential to elucidate further unique gradients in PPS boundary representations, thus providing insight on the categorization and function of shallow versus sharp boundaries (Noel et al., 2017).

Finally, studying the neural correlates of PPS in diverse contexts (e.g., under threat, in social context) among healthy individuals and individuals with trauma-related disorders appears crucial to a deeper understanding of functional connectivity and integration between subcortical structures in the brainstem that are dedicated to innate alarm response, balance, and consciousness, sensorimotor cortices devoted to multisensory integration, and prefrontal and posterior cortices involved in higher-order cognition, such as social cognition and self-referential processing.

## Author Contributions

DR contributed to conception, literature review, and wrote the original draft of the manuscript. MM and RL contributed to funding acquisition. All authors contributed to manuscript revision, read, and approved the submitted version.

## Conflict of Interest

The authors declare that the research was conducted in the absence of any commercial or financial relationships that could be construed as a potential conflict of interest.

## Footnotes

1 The visuo-tactile interference (VTI) paradigm requires study participants to discriminate between tactile stimuli delivered at different locations (up or down) on the participant’s finger while task-irrelevant visual stimuli are displayed on a target object. When investigating VTI during movement, study participants were asked to perform the task (discriminating the location of a tactile stimulus) while grasping the target object (Brozzoli et al., 2011).
